# Health system support and health system strengthening: two key facilitators to the implementation of ambulatory tuberculosis treatment in Uzbekistan

**DOI:** 10.1186/s13561-016-0100-z

**Published:** 2016-07-12

**Authors:** Stefan Kohler, Damin Abdurakhimovich Asadov, Andreas Bründer, Sean Healy, Atadjan Karimovich Khamraev, Natalia Sergeeva, Peter Tinnemann

**Affiliations:** 1 Institute of Public Health, Heidelberg University, Heidelberg, Germany; 2 Institute for Social Medicine, Epidemiology and Health Economics, Charité – Universitätsmedizin Berlin, Berlin, Germany; 3 Médecins Sans Frontières, Nukus and Tashkent, Uzbekistan; 4 Department of Health Management, Evidence-based Medicine Centre, Tashkent Institute of Postgraduate Medical Education, Tashkent, Uzbekistan; 5 Tashkent Pediatric Medical Institute, Nukus Branch, Nukus, Uzbekistan

**Keywords:** Ambulatory care, Financing, Health system, Hospitalization, Outpatient care, Scale-up, Stakeholder perceptions, Tuberculosis, Uzbekistan

## Abstract

Uzbekistan inherited a hospital-based health system from the Soviet Union. We explore the health system-related challenges faced during the scale-up of ambulatory (outpatient) treatment for drug-susceptible and drug-resistant tuberculosis (TB) in Karakalpakstan in Uzbekistan. Semi-structured interviews were conducted with key informants of the TB services, the ministries of health and finance, and their TB control partners. Structural challenges and resource needs were both discussed as obstacles to the expansion of ambulatory TB treatment. Respondents stated need for revising the financing mechanisms of the TB services to incentivize referral to ambulatory TB treatment. An increased workload and need for transportation in ambulatory TB care were also pointed out by respondents, given the quickly rising outpatient numbers but per capita financing of outpatient care. Policy makers showed strong interest in good practice examples for financing ambulatory-based management of TB in comparable contexts and in guidance for revising the financing of the TB services in a way that strengthens ambulatory TB treatment. To facilitate changing the model of care, TB control strategies emphasizing ambulatory care in hospital-oriented health systems should anticipate health system support and strengthening needs, and provide a plan of action to resolve both. Addressing both types of needs may require not only involving TB control and health financing actors, but also increasing knowledge about viable and tested financing mechanisms that incentivize the adoption of new models of care for TB.

## Introduction

Ambulatory-based management of tuberculosis (TB), including multidrug-resistant tuberculosis (MDR-TB), appears to be effective and cost-effective in a variety of settings [1–6]. With potential to increase the cost-effective use of resources and to reduce the lag between diagnosis and start of treatment, ambulatory treatment of drug-susceptible as well as drug-resistant TB can help improve quick and universal access to TB treatment, which in turn mitigates the spread of TB in the population. In addition, ambulatory treatment can reduce the risk of nosocomial TB transmission to hospital staff and among patients, and it enables patients to reduce the costs associated with reduced time at work or home [7–9]. The World Health Organization (WHO) has encouraged outpatient TB management in resource-limited settings since the '90s [10] and conditionally recommends ambulatory treatment of MDR-TB since 2011 [11]. The WHO reiterated these recommendations in its post-2015 global TB strategy, labelled the End TB Strategy [12], and has advised ambulatory-based management of MDR-TB in all post-2011 Global Tuberculosis Reports [13–16]:

*“Ambulatory services should be given preference over hospitalization, which should be limited to severe cases.” (WHO End TB Strategy)*

*“Greater use of ambulatory care as part of decentralized PMDT [programmatic management of drug-resistant TB] services is necessary to expand access.” (WHO Global Tuberculosis Report 2015)*

For the WHO European Region, a new TB action plan was adopted in September 2015. It states that all WHO high-priority countries for TB control in the European region should specify strategies and mechanisms for expanding and maintaining ambulatory TB treatment by 2016 [17]. Presently, the treatment practices for TB involve high levels of hospitalization in several high MDR-TB burden countries. The hospitalization level of MDR-TB patients (at least for part of the treatment) was between 75 and 100 % in Eastern European countries, with the lowest levels in Central Asia (30–40 % in Kazakhstan, Tajikistan and Uzbekistan). The hospitalization level varied widely in the African Region (21 % of patients in the Democratic Republic of the Congo to 100 % in Nigeria) [15]. Hospitalization for 100 % of MDR-TB patients in 2013 was reported for India, China and the Russian Federation, the three highest MDR-TB burden countries in the world, and in 2014 for China and the Russian Federation together with 8 other high MDR-TB burden countries [15, 16]. Hence, many more TB patients could and, based on the preliminary evidence on effectiveness and cost-effectiveness of ambulatory MDR-TB treatment [2-6], probably should be treated using ambulatory-based care rather than hospital-based care in several high MDR-TB burden countries.

Uzbekistan has reduced the number of TB beds from more than 15,000 in 2008 to less than 11,000 in 2012, and decreased the duration of hospitalization for MDR-TB patients from 270 to 90 days during the same period [13]. In 2011, *Presidential Decree No. 1652* [18] ordered a further reduction of the number of state-funded hospital beds, including TB beds. The decree instructed a decrease in the number of buildings in the tuberculosis care network from 152 (pre-2012) to 76, once fully implemented [18, 19]. Notwithstanding this nationwide reduction in TB-bed capacity and the inclusion of ambulatory-based TB care in the Uzbek strategy to achieve universal access to TB treatment [20], the treatment practice of TB remained hospital-based until October 2014 in all parts of Uzbekistan except for two regions with pilot programs for ambulatory-based TB care, the Republic of Karakalpakstan and Tashkent city. The reason was *Decree No. 160* and other *prikazes* (decrees) of the Ministry of Health (MoH) of Uzbekistan, which required the treatment of all TB patients, other than exceptional cases, to start in a TB hospital [21].

Starting as well as continuing TB treatment on an ambulatory basis was permitted nationwide throughout Uzbekistan on October 24, 2014, when the MoH of Uzbekistan issued *Decree No. 383* “On improvement of TB care activities in the Republic of Uzbekistan” [22]. *Decree No. 383* specifies groups of TB patients to be selected for ambulatory-based treatment. These groups consist of drug-susceptible and drug-resistant TB patients with negative sputum smear status whose condition does not require continuous monitoring, TB patients with uncomplicated active extrapulmonary TB, TB-infected children without risk factors and, with permission of a medical expert commission, exceptional cases in which a sputum smear positive TB patient wishes ambulatory treatment. In addition, the decree states that sputum smear positive TB patient may be treated home-based if adherence as well as specified criteria for uninterrupted treatment and infection control can be assured.

In Karakalpakstan, a republic in the northwestern part of Uzbekistan, ambulatory treatment from day one has been offered to patients with drug-susceptible and drug-resistant forms of TB, including MDR-TB, already since February 2011. The MoH of Karakalpakstan and Médecins Sans Frontières (MSF) began implementing a program, called “Comprehensive TB Care for All” [8], which introduced rapid testing of drug-resistance and integrated directly observed treatment, short-course (DOTS) and DOTS-Plus treatment into a common ambulatory TB care strategy for drug-susceptible and MDR-TB patients. The deviation from the national regulation that, until October 2014, required hospitalization for at least part of any TB treatment was made possible through *Decree No. 39* of the MoH of Karakalpakstan, which gave permission for ambulatory treatment of drug-susceptible and drug-resistant TB from the day of diagnosis onward, unless a patient’s poor health or socio-hygienic living conditions require hospitalization [23]. The aim of the “Comprehensive TB Care for All” program is to scale-up the ambulatory TB care model until universal access to TB treatment in Karakalpakstan is achieved [24].

Despite having established the regulatory basis for the expansion of ambulatory treatment of drug-susceptible and drug-resistant TB the incentives set by the Uzbek health system, which finances hospitals based on their bed occupancy, have been perceived to work against a fast and full adoption of ambulatory-based TB care in Karakalpakstan. The experience of this setback has been described in an MSF information booklet about the “Comprehensive TB Care for All” program already at an early stage of implementation [8]:

*“Ambulatory [TB] care should be offered to all patients from the very start of treatment, unless there are specific medical indications requiring admission. Ambulatory treatment should generally be preferred to hospital-based treatment because it reduces the chance of cross-infection of hospitalized patients with drug-resistant strains, and because it could likely reduce adherence problems related to prolonged hospital stays, such as the isolation of the patient from their social environment. […] Presently, health system financing in Uzbekistan is based on bed occupancy, on the “per number of beds” principle. This creates incentives for keeping patients in hospital for longer than is necessary, which is exactly the opposite of the desired situation. Instead, a system needs to be introduced, with advice from appropriate experts in health-system financing, which provides a proper mix of incentives for good-quality treatment.” (MSF information booklet “Comprehensive TB Care for All”)*

The described problem of medically unnecessary hospital stays is known as overhospitalization. It has been discussed as an undesirable effect, in particular, in health systems which once created a large hospital capacity, such as those of former Soviet Union countries [19, 25]. While the general health sector has been and continues to be reformed in several former Soviet Republics since their independence, specialized health services, such as the TB care sector in Uzbekistan, have usually not been part of past health reforms and continue to work in the traditional way with a few exceptions [26–28]. Unsurprisingly, overhospitalization of TB patients has been described previously for post-Soviet states, for instance, after ambulatory DOTS programs were introduced in Russia [29, 30] or after the Stop TB strategy was implemented in Armenia [31, 32].

Based on the Karakalpakstan experience, this study illustrates how the traditional financing mechanisms of the TB services in the post-Soviet health system of Uzbekistan are perceived as disincentives to the adoption of a comprehensive ambulatory care model for drug-susceptible and drug-resistant TB. The study also shows that the perceived challenges to scale-up of ambulatory TB treatment fit into a classification of health system support and health system strengthening needs. The health system strengthening and support needs described by the study participants during individual and group interviews exemplify the range of challenges that can pose barriers to adopting comprehensive ambulatory-based management of TB as recommended by the WHO.

## Background

### TB and TB care in Karakalpakstan, Uzbekistan

Some of Uzbekistan’s highest TB burden occurs in its northwestern part, the Republic of Karakalpakstan with a population of 1.8 million people. In 2013, the TB prevalence in this region was 107.7 per 100,000 population and the TB mortality was 10 per 100,000 population according to MoH data. Multidrug-resistance rates were 40.8 % among new and 78.1 % among retreated TB cases in 2010 [33].

The TB services are a specialized health service in Karakalpakstan. They are governed by similar principles as the Uzbek health system, which has evolved from the Soviet Semashko model of health care. The government-owned health system of Uzbekistan has been described as strictly hierarchical, using predominantly policy formulation as a mode of regulation. Subordinate levels of the health system are expected to follow the policies set by higher levels. Neither fiscal nor other forms of incentives are traditionally used for the regulation of health care providers [19, 34].

The TB services are budgeted by the treasury and operated through the MoH. In the ambulatory sector, primary health care facilities are funded based on the population of their catchment area, taking into account its age and gender composition. Consequently, health facilities do not receive additional financial resources that are directly tied to the growing number of outpatients during the expansion of ambulatory TB care. Given the per capita financing of outpatient care, the increasing outpatient numbers resulted in an availability of fewer resources per patient and a higher workload for the staff of the ambulatory TB services, despite an indexed budget that has grown. Hospital financing is based on the annual average of inpatient bed numbers and bed occupancy rates. Due to the bed-based funding, TB hospitals that refer patients to ambulatory treatment risk budget cuts. Past government decrees endorsed comprehensive ambulatory TB treatment and decreased the number of TB hospitals and beds available in Karakalpakstan [23, 35], but no regulation changed the system-inherent incentives to hospitalize TB patients. The regulation and financing mechanisms of the TB services in Karakalpakstan and the incentives they set are discussed in more detail elsewhere [27, 36] and are similar to those described for Armenia prior to 2014 [26].

The TB services in Karakalpakstan have been collaborating with MSF since 1998 to improve and expand TB treatment. Initially a DOTS program was implemented as a TB control strategy. The DOTS program had covered all districts in Karakalpakstan until August 2003. Starting in September 2003, a DOTS-Plus program added components for MDR-TB diagnosis, management and treatment. All patients diagnosed with active drug-susceptible TB and MDR-TB were admitted for an *intensive phase* of at least 2 and 8 months of inpatient treatment, respectively, or longer if needed to achieve sputum conversion. The subsequent *continuation phase* of treatment was administered through special DOTS corners in outpatient clinics that only treated MDR-TB patients. In the course of the scale-up of the DOTS-Plus program, the management of drug-susceptible and drug-resistant TB was integrated for the *continuation phase*. Case detection, disease management and treatment were fully integrated for drug-susceptible and drug-resistant TB in 2010 (“Comprehensive TB Care for All”). Since February 2011, *Decree No. 39* of the MoH Karakalpakstan permits ambulatory intensive phase treatment of drug-susceptible and drug-resistant TB, and to hospitalize selectively only those patients whose clinical condition or living conditions require hospitalization [23]. Table 1 provides a chronology of the TB control program in Karakalpakstan.

**Table 1:** Chronology of the TB control program in Karakalpakstan, Uzbekistan

Date	Activity
July 1998	First patient entered in DOTS program in Muynak district
June 2001	Launch of DST study
July 2002	First staff appointed to MDR-TB program (MDR-TB medical doctor position)
April 2003	Ministry of Health of the Republic of Uzbekistan issued *Decree No. 160* on management of TB [21]
August 2003	DOTS expanded to the last uncovered district Turtkul
September 2003	Start of DOTS-Plus program. First MDR-TB patients admitted to Republican TB Hospital No. 2 in Nukus
October 2004	Outpatient department opens in Nukus city for ambulatory care of MDR-TB patients discharged from MDR-TB hospital
May 2005	First MDR-TB patient cured
September 2005	Signed memorandum of understanding with Foundation for Innovative New Diagnostics for rapid DST
January 2006	GFATM TB program starts to supply second-line TB drugs for Karakalpakstan
October 2007	Ministry of Health of the Republic of Karakalpakstan issued *Decree No. 366* on expansion of ambulatory care of drug-resistant TB through early discharge into ambulatory care
May 2008	Ministry of Health of the Republic of Uzbekistan issued *Decree No. 180* on management of MDR-TB
August 2010	“Comprehensive TB Care for All” program approved in Karakalpakstan
February 2011	Ministry of Health of the Republic of Karakalpakstan issued *Decree No. 39* on “Comprehensive TB Care for All” program expansion and management of TB [23]
March 2011	Cabinet of Ministers of the Republic of Uzbekistan issued *Decree No. 62* on construction, reconstruction and refurbishment of TB facilities, and optimization of inpatient facilities through abolition of low capacity inefficient TB facilities [35]
December 2015	“Comprehensive TB Care for All” program implemented in all districts

*DOTS* directly observed treatment, short-course; *DOTS-Plus* is a management strategy for MDR-TB built upon the elements of DOTS, *DST* drug susceptibility testing, *GFATM* Global Fund to Fight AIDS, Tuberculosis and Malaria, *MDR-TB* multidrug-resistant tuberculosis, *MSF* Médecins Sans Frontières. Source: WHO [56] and own compilation

The TB bed capacity in Karakalpakstan was reduced by more than 20 % (320 beds) from 2010 to 2014 based on *Decree No. 62* of the Cabinet of Ministers of the Government of the Republic of Uzbekistan entitled “On additional measures to decrease the incidence of tuberculosis in the Republic of Uzbekistan for 2011-2015” [35]. Savings achieved by the downsizing of the inpatient TB services were reinvested in the refurbishment and modernization of the equipment of the TB hospitals maintained. The MoH of Karakalpakstan plans to further reduce the available TB hospital capacity, but the MoH cannot reinvest savings from the inpatient TB services into the ambulatory TB services under the financing mechanisms in place.

Ambulatory TB care is offered through DOTs corners in polyclinics, primary health care facilities (*Selskie Vrachebniye Punkti*) and local health posts (former *Feldsher-Accoucheur points*) that operate as branches of the primary health care facilities. In 2014, inpatient TB care in the districts of Karakalpakstan was offered in seven specialized TB hospitals, comprising *rayon* (district) and inter-*rayon* hospitals. In addition, two Republican TB Hospitals (No. 1 and No. 2) remained in Nukus City after the closure of a third Republican TB Hospital (No. 3). Each TB hospital has a smear positive and smear negative ward, but only the Republican TB Hospitals in Nukus City and an inter-*rayon* TB hospital in Chimbay treat drug-resistant TB. Average costs of a hospital bed in Nukus City in 2013 for a drug-susceptible TB patient were UZS 30,169 (USD 15) per day and UZS 1,206,756 UZS (USD 603) per case treated. The average length of stay was 40 days for drug-susceptible patients and 100 days for patients with drug-resistant TB [9]. Cost or cost-effectiveness estimates comparing ambulatory and hospital-based treatment of MDR-TB in Karakalpakstan are not available.

Before the “Comprehensive TB Care for All” program, all MDR-TB patients had their treatment initiated in Republican TB Hospital No. 1 or No. 2. In districts without a TB inpatient department, patients were referred to the regional capital city Nukus or to the closest district which has one. Patients receiving immediate ambulatory treatment typically start their treatment in a TB cabinet room and are then referred to the local outpatient primary health care facility. There is usually a TB cabinet room or TB dispensary (a specialized ambulatory facility) in all districts. It is regularly attended by a TB doctor, who is the only medical specialist with the authority to establish an official diagnosis of TB.

Looking at the economic context, Uzbekistan has faced steady economic growth over the past decade, and government expenditures on health were predicted to rise from 2.9 % of the GDP in 2010 to 4.4 % of the GDP in 2020 [37]. The budget of the TB services has also steadily increased over the past few years, with an annual increase of approximately 30 %.

### Cost and cost-effectiveness of treatment for MDR-TB

The cost per MDR-TB patient treated in Uzbekistan in 2014 was reported to be USD 2935 [16]. One systematic review identified four studies on the cost and cost-effectiveness of MDR-TB treatment in other countries [3]. From the health system perspective, the cost (year 2005 USD) per disability-adjusted life-year (DALY) averted through ambulatory-based management of MDR-TB was estimated at USD 163 in Peru and USD 143 in the Philippines (no and 7 days average hospitalization) compared to USD 598 in Estonia and USD 745 in Tomsk. In Estonia and Tomsk, treatment was hospital-oriented with, on average, 192 days and 239 days of hospitalization, respectively. The cost per patient for MDR-TB treatment was USD 2423, USD 3613, USD 10,880 and USD 14,657, respectively. Synthesizing the data from these four studies and using probabilistic sensitivity analysis, the systematic review also appraised the likely cost and cost-effectiveness of MDR-TB treatment for 14 WHO subregions (covering 193 countries). Based on the model predictions, treatment of MDR-TB in either model of care appeared highly cost-effective in all 14 WHO subregions considered, according to the WHO threshold that a health care intervention is highly cost-effective if it costs less than the annual GDP per capita per DALY averted [38]. The usefulness of the WHO cost-effectiveness thresholds as a guide for policy-makers is under debate [39], but the four studies reviewed and the model predictions for the WHO subregions further indicated a better cost-effectiveness for outpatient versus inpatient models of care.

For the WHO subregion EUR B, which had an average GDP per capita of USD 3384 and includes the Central Asian republics Kyrgyzstan, Tajikistan, Turkmenistan and Uzbekistan, cost per DALY averted were estimated to be USD 316 (123–672) per patient treated ambulatory and USD 801 (371–1,571) per patient treated hospital-based. Cost per patient treated were estimated to be USD 6,057 (2,955–11,692) in the outpatient model and USD 15,505 (8,063–29,015) in the inpatient model, respectively. These estimates from the probabilistic sensitivity analysis represent means with 5th and 95th percentiles of the cost distributions. The systematic review and other studies note that the main influences on cost are the model of care and the drugs included in the treatment regimen [3, 40, 41]. For patients, income loss often constitutes the largest financial risk from TB [41, 42].

### Health reform in post-Soviet states and financing of TB services

There is a substantial body of literature on health reform in Central Asia (Kazakhstan, Kyrgyzstan, Tajikistan, Turkmenistan and Uzbekistan) and other regions of the former Soviet Union [43–47]. Several post-Soviet states have reformed the provision of services in primary health care, and they have reduced the number of hospital beds since independence. With a few exceptions, the funding mechanisms for the specialized TB services remained unchanged. In Armenia, new financing mechanisms for both inpatient and outpatient TB services were introduced in 2014, along with new criteria for hospital admission and discharge. Bed-based financing for the inpatient TB services has been replaced by fixed and variable cost funding. Per-capita financing of the outpatient TB services has been replaced by performance-based funding. The new funding scheme also allows money saved to be reinvested into an outpatient oriented TB program and aims to produce a gradual shift of experienced TB doctors from hospitals to outpatient services [26, 27]. Belarus has started a pilot project in 2014, under which money that became available after a reduction in the number of beds in one TB hospital would be used to incentivize primary health care workers providing DOTS to rural outpatients [27]. Beginning in 2011, the TB services have been reformed and integrated into the primary health care in Georgia [28].

### Theoretical framework

Supporting the health system can include any activity that improves services. Support activities improve outcomes primarily by increasing inputs. By contrast, strengthening the health system is accomplished by more comprehensive changes to policies and regulations, organizational structures and relationships across the health system building blocks. Strengthening activities motivate changes in behavior and/or allow a more effective use of resources to improve (multiple) health services. Hence, health system strengthening is about making the system function better permanently, not just filling gaps or supporting the system to produce better short-term outcomes [48].

A wide spectrum of health system strengthening interpretations exists. We adopted criteria suggested by Chee et al. [48] in order to distinguish between health system strengthening and support. If the questions in Table 2 could be answered with “Yes”, then coded interview content that related to a potentially needed activity or intervention was classified as a health system strengthening need, otherwise coded content was identified as a health system support need.

**Table 2:** Is it Health System Strengthening?

Criteria to distinguish between health system strengthening and support interventions	
1.	Do the interventions address policy and organizational constraints or strengthen relationships between the six WHO [57] building blocks of a health system (service delivery, health workforce, information, medical products, vaccines and technologies, financing, and leadership and governance)?
2.	Will the interventions produce permanent systemic impact beyond the term of the project?
3.	Are the interventions tailored to country-specific constraints and opportunities, with clearly defined roles for country institutions?

Source: Adapted from Chee et al. [48]

Chee et al. [48] also require a fourth question to be asked: “Do the interventions have cross-cutting benefits beyond a single disease?” We did not apply this fourth criterion because our study focused on the TB services, which are organized and funded as a separate health service with a disease-specific mandate within the general health system in Uzbekistan.

According to Chee at al. [48] not distinguishing health system support activities from strengthening ones can lead to neglect of critical system strengthening activities, as well as to unmet expectations of stronger health systems. They therefore argue that distinguishing between these two types of activities will improve programming impact.

## Methods

### Study setting and design

While making progress towards changing the model of care for TB toward a decentralized ambulatory approach since February 2011, MSF and the MoH of Krakalpakstan have noted that the existing health system financing fails to incentivize the use of ambulatory-based TB care. In response, this exploratory qualitative study was implemented aiming to assess the policy for allocation of funds for TB care in Karakalpakstan, the ways in which it undermines the implementation of decentralized ambulatory care, and how the health financing could be changed to support the expansion of ambulatory care for drug-susceptible and drug-resistant TB.

Interviews with one respondent and interviews with several respondents were conducted between September and October 2012 in Tashkent as well as in Nukus and two districts of Karakalpakstan. Arrangements were made to meet at a workplace convenient for all the participants in order for the interviews to be conducted. During a typical interview, the questions progressed from asking about the TB care financing in place, through perceived challenges with respect to implementing ambulatory TB care, to possible suggestions and solutions. Open questions that subsequently became more specific were asked within each topic. No prespecified set of questions was used throughout due to the various, usually natural settings in which interviews took place.

### Study participants

Key informants with a high level of experience in the provision, organization or financing of the TB services in Karakalpakstan and/or the rest of Uzbekistan were identified and purposively contacted by staff of the Uzbek mission of MSF who were familiar with the potential participants. To learn about the views and perception of various stakeholders, participants were selected from the health and finance ministries, the TB services in Karakalpakstan and from TB control partners (Global Fund to Fight AIDS, Tuberculosis and Malaria; MSF, Project Hope). A total of 24 unique respondents (including authors AB, AK and NS) participated in 15 interviews. An interview with more than one respondent was conducted when more participants from the same group of participants could be met together. Group sizes varied from two to five respondents. Two respondents participated in a one-on-one interview and a group interview (Table 3).

**Table 3:** Study participants

Type of participant	Affiliation	Level	Tool
Government official or employee	Ministry of Health	Uzbekistan	Individual interview
	Ministry of Health, Treasury or government	Karakalpakstan	Individual interview^a^
			2 x Group interview^a^ (2 participants per group)
TB care provider	TB services (inpatient and outpatient)	Karakalpakstan	2 x Individual interview
			4 x Group interview^b^ (2–3 participants per group)
TB control partner	GFTMA	Uzbekistan	Individual interview
	Project Hope	Uzbekistan	Individual interview
	MSF^c^	Karakalpakstan and Uzbekistan	2 x Individual interview^d^
			Group interview^d^ (5 participants)

*MSF* Médecins Sans Frontières, *GFATM* Global Fund to Fight AIDS, Tuberculosis and Malaria, *TB* tuberculosis

^a,d^One respondent participated in individual and group interview

^b^Included one city health department staff

^c^Included local and international MSF staff

### Data collection

SK conducted all interviews. Interviews were either audio recorded or written summary notes were taken by SK. The majority of the conversations lasted between 45 minutes and 2 hours; two interviews lasted approximately 15 minutes. Interviews usually took place at the respondents’ place of work, or, in one case, during a work-related commute between health facilities. The commute was chosen on purpose for one group interview with TB doctors because the commute constituted a calm time among peers during their work day. In eleven cases, an interview was conducted in a local language and translation was needed. Translation was provided by local MSF staff. A translator was only present if translation was needed. In five cases, one or two familiar people, such as colleagues or MSF staff, were present but were not involved in the core conversation. The number of discussions was not predetermined. Data collection continued until the ideas expressed were repetitions of concepts already identified, that is, data saturation had been reached.

### Data management and analysis

The data collected in individual and group interviews were analyzed in the same manner using thematic analysis, a method for identifying, analyzing and reporting patterns within qualitative data. The analysis was based on organizing sections of the data into recurrent themes and using quotes to illustrate the kind of data classified within each theme [49, 50]. Data analysis began after the first interview. The analysis was an ongoing process. Recordings were listened to and notes were read through several times to obtain a sense of the whole. Relevant parts of the audio recorded English conversations or the simultaneous English translations were transcribed verbatim into written form using a text editor.

The data set used for the analysis was all text extracted from the recording and notes. The analyst used codes to identify explicit content that appeared interesting to him regarding (financial) resources and incentives in TB care. Reflecting on the pattern in the data, codes were collated into potential themes. Thinking about the relationship between themes, different levels of themes were divided into main overarching themes and subthemes within them. The analyst reviewed and refined the potential subthemes and themes until he devised a satisfactory thematic map, taking into account his review of related literature and key documents. Finally, the analyst checked whether the themes identified work in relation to the coded extracts as well as the entire dataset. The theoretical framework by Chee et al. [48] to distinguish health system strengthening and support activities was used in the derivation of the final themes. All management and analysis was performed by SK.

### Permissions

Permission to conduct the study was granted by the MSF desk in Berlin and the MSF mission in Uzbekistan. Potential study participants were contacted with information about the study. An interview was scheduled with those who agreed and were available to participate. Interviews were recorded if permission was given and the setting was suitable for recording. An opportunity to revise the quotes reproduced was granted. Written consent to reproduce the selected anonymized quotes was obtained.

## Results

A wide range of issues relating to the implementation and expansion of ambulatory TB care was discussed. Interview content that related to the financing of ambulatory TB care and its expansion was finally stratified into three main themes: health system strengthening, health system support and resources available. The focal theme, health system strengthening, was further stratified into two subthemes: financial disincentives to ambulatory TB treatment and ideas for and enablers of change.

### Health system strengthening

#### Financial disincentives to ambulatory TB treatment

Respondents reported the transition from hospital-based TB care to ambulatory treatment is proving difficult, given the financing of the TB services in Karakalpakstan. Three major financial disincentives that hinder the scale-up of ambulatory treatment were expressed.

Firstly, respondents explained that the funding of outpatient TB care facilities is based on the population size, age and sex composition of the area served. The resources available for ambulatory TB care were perceived as unresponsive to the increasing outpatient numbers experienced during the scale-up. Decreasing resources per patient and a higher workload for the staff of the ambulatory TB services due to expanding outpatient treatment of TB were described.

Secondly, the traditional bed-based financing of TB hospitals has been perceived to undermine the transition to mainly ambulatory TB treatment. The hospital financing in place was described as a reason for continuingly high proportions of inpatient TB treatment, despite the new possibility to treat drug-susceptible and drug-resistant TB patients as outpatients from the day of diagnosis in the “Comprehensive TB Care for All” program districts.

*“Going back to the beginning of last year, 2011, when we got the approval from Karakalpakistan that we could treat patients from day 1 on ambulatory care, then shortly afterwards we realized there are two forces which are working against each other. This issue of how the government is financing the system became so obvious. One force was that there is this prikaz [MoH of Karakalpakstan Decree No. 39] which pushes people into ambulatory care from day 1, so they are not coming to the hospital. And the other force goes against this, but you have to fill up your beds in the hospital. So on the first day the doctors were agreeing to treat the patient in an ambulatory way, and the next day they grab the same patient, the same doctors grab the same patient back into the hospital to fill up the beds, because that is how they get the money.” (Group interview 1)*

*“The past practice was to finance the inpatient facilities’ activities based on the number of the beds. And one of the indicators of the medical facility activity is to fulfill the plan of bed occupancy. In such cases, you have to keep some patients longer than necessary and not to discharge them from the hospital. It is, therefore, the right decision to discuss the issue of financing based on the number of patients. So, irrespectively of where a patient is receiving his treatment, the financial amount assigned for his treatment will follow him.” (Interview 4)*

Thirdly, respondents described separate budgets for inpatient and outpatient TB services, and that funds allocated to inpatient TB care, including savings from TB bed reductions, cannot be reallocated by the MoH to the expansion of ambulatory TB care. One respondent further pointed out that the failure of the TB care financing to reward successful treatment can also contribute to a high rate of hospitalization of TB patients in Karakalpakstan:

*“The treatment outcome does not really affect the financing. So, it is a slight problem because if the facility gets the funding according to the number of the treated, like cured patients, then more focus would be on the treatment outcome, successful outcome. It is either cured or treatment completed. But as such an approach does not work, so the facility is only interested in having the beds occupied. Someone goes, another one comes and fills in the bed. So it is not a treatment outcome-oriented or patient-oriented approach at all. So it is about occupying your beds.” (Interview 2)*

#### Ideas for and enablers of change

While disincentives to the implementation of ambulatory TB treatment were repeatedly described in precise and elaborate ways, only a few respondents put forward ideas for alternative TB care financing mechanisms and their suitability for the local context. Prompting for viable options to modify the TB care financing in place, case-based financing mechanisms, pay for performance that gives financial incentives for better health outcomes, and remuneration that is adjusted for the caseload and the complexity of cases were all suggested as possible elements of an alternative to the current funding of the TB services. These ideas were expressed without an implementation plan, for instance, describing that financing of TB services should be for the actual number of cases treated, should take into account treatment outcomes or that salary should be according to the difficulty of the job and depending on the number of patients.

Respondents were interested in training and real-life examples on how to strengthen ambulatory TB care as a specialized service in their health system. They indicated that it would be useful to learn from the experience of other countries, in particular post-Soviet states, which have implemented financing reforms for their TB services.

*“Because if it comes out and the state does not have a real proof that it works, they may not change anything, in the financing either. So I think they need proof at a very high level like consultancy that it has worked in similar settings, like other post-soviet countries, because now almost everywhere the system is unchanged.” (Interview 2)*

### Health system support

The focus of the interviews conducted was to elicit structural barriers in the health system that work against the scale-up of ambulatory TB treatment. Nevertheless, immediate resource needs also came up during the conversations, particularly if respondents were involved in the day-to-day provision rather than the management or organization of the TB services. The main needs reported included a lack of ambulatory staff, due to an increased workload resulting from the scale-up of ambulatory treatment, and a lack of transport capacity between facilities. Some respondents said that they knew of ambulatory staff in the TB services who had paid out of their own pocket for transportation or work-related phone calls in the past because the resources provided were sometimes insufficient.

### Resources available

Responses in our study indicated an existing awareness that health system strengthening needs to accompany health system support within the TB services. In addition, there appeared to be willingness to change the hospital-based system and respondents shared a positive attitude toward ambulatory TB treatment.

*“There is certain interest in outpatient treatment from all stakeholders, including the national partners. We hear more and more about the possibility and necessity to go more for outpatient care. Levels as high as the ministry would also say the same. They are looking for such a change, and they are looking for the consultants to advise them on this, how to go through this reform. This is the situation now.” (Interview 2)*

## Discussion

Most former Soviet Union countries have strong roots in providing hospital-based TB care, and they had often created a large capacity of hospital beds in a centralized health care system. The traditional financing mechanisms of the TB services, which continued to be in place in Uzbekistan and other post-Soviet states after the dissolution of the Soviet Union, provide no incentives for earliest possible referral to ambulatory care and fail to reallocate resources towards the expansion of ambulatory TB treatment [36]. Interview participants in this qualitative study in the context of the expansion of ambulatory treatment for drug-susceptible and drug-resistant TB in Karakalpakstan described a range of health system support and strengthening needs that were perceived as barriers to changing the model of TB care.

The health system strengthening needs for the TB services, which were enumerated during interviews, resembles the common health system challenges of post-Soviet Union states after their independence. Firstly, a substantial proportion of medical treatment takes place in hospitals that are financed based on bed numbers and occupancy rates, despite the fact that several procedures could probably be done efficiently and cost-effectively in an ambulatory setting. Secondly, the ambulatory sector needed for comprehensive outpatient care is underdeveloped and underfinanced [19, 46]. A further aspect related to overhospitalization is specific to TB care, namely that hospitalization of TB patients without sufficient isolation poses a threat to the patients’ and medical staff’s health as it may cause infection with more resistant strains of TB during treatment in hospital [51].

In the study context of Karakalpakstan, the stakeholders involved in the organization and provision of the TB services appeared committed and willing to change the present TB care financing in order to implement and scale-up ambulatory TB care. Positive attitudes of key stakeholders toward change help implement new TB treatment strategies and may increase the pace of change and the sustainability of change when achieved [52]. However, the local public health decision-makers interviewed described that lack of relevant evidence, best practice examples or expert advice on desirable TB care financing mechanisms hinders reforming the financing of the TB services.

Few past health system strengthening activities have focused specifically on revising the traditional financing mechanisms of the TB services in former Soviet states [26, 27], despite the fact that these financing mechanisms work against the adoption of ambulatory TB treatment approaches. This may reflect that further operational and health systems research for improving the performance and introduction of new TB care delivery strategies is needed [53]. As it may not be feasible to archive a reform focused specifically on the financing mechanisms of the TB services, health financing reform addressing inefficiencies, like overhospitalization, may need to be sought and adopted in a broader context, taking into account specialized health sectors, such as the TB services in many post-Soviet countries.

Health system changes that support the ambulatory treatment model have already been achieved in Karakalpakstan in an area other than financing of the TB services. As ambulatory treatment of TB patients uses more decentralized structures than inpatient treatment, the clinical management has been decentralized. Traditionally, every decision for each drug-susceptible and drug-resistant TB patient went to a single *consilium*, the medical expert commission authorized to make TB treatment decisions, where each case is debated by specialists. Meanwhile, most cases are reviewed by several *mini consilia* within the districts. However, achieving this reform in the health system has been described as difficult in an MSF information booklet about the scale-up of ambulatory TB treatment [24]:

*“Since 2011 there has been significant system change, although achieving this has been a lengthy process requiring the devolution of the decision making process on TB diagnosis and care from a single, centralised consilium to district level.” (MSF information booklet “The Path to Scale-up”)*

## Strengths and limitations

The results of this study are subject to several limitations. Some informants on the national level were unavailable due to a busy working schedule. Not all interviews were audio recorded and only notes were taken. The variety of stakeholders interviewed facilitated broad rather than specific insights. Responses frequently involved centralized decision-making and top-down regulation as a driver of change, possibly due to the predominant experience of the study participants with the present system. Translators were experienced with medical translation, but provided real-time translation on partially new topics. In some instances, the information and quotations translated may, therefore, reflect content summaries rather than actual wording. Interviewing more than one person at the same time may have reduced the diversity of opinions. On the other hand, interviewing a group can supplement interviewing individuals, and it could have assisted respondents to reevaluate a previous position or statement in need of “amplification, qualification, amendment or contradiction” [54]. We acknowledge that the variation in interviewing methods limits the scope for understanding their individual strengths and limitations in our study context.

A coherent picture emerged across the individual interviews and group interviews, but our findings may be biased by the small study sample and lack of independent data. Three interview participants have been involved in the conception and design of the study and contributed to writing the manuscript. None of them has been involved in data analysis and management. Finally, the interview partners were a selected group of respondents with close ties to each other, many of whom were involved in TB care and the scale-up of ambulatory TB treatment. By contrast, the familiarity among the relatively small number of key people involved with TB care in Karakalpakstan ensured an in-depth coverage of the local situation. We, therefore, believe that the insights gained from this study are representative of the health system strengthening and support needs experienced by the TB services in Karakalpakstan in the course of the scale-up of ambulatory-based management of TB.

## Conclusions

Health systems may receive substantial support for effective TB control, but complementary health system strengthening can help to make adjustments to incentivize the adoption of new treatment approaches, such as comprehensive ambulatory-based care of patients with drug-susceptible and drug-resistant TB. The findings of this study show that health system strengthening has been perceived to be necessary for implementing and expanding ambulatory TB treatment in Karakalpakstan, Uzbekistan, notwithstanding the support received or requested. The experience of Karakalpakstan illustrates the range of factors that may need to be considered to develop an effective TB control strategy in post-Semashko health systems, and that further research on improving the introduction of new TB care delivery strategies is needed.

To facilitate the adoption of ambulatory models of care, TB control strategies should anticipate both health system support and strengthening needs, and the possible consequence of involving health and finance ministries, as well as TB control and health financing reform partners in the process of restructuring the model of care for TB. Anticipating health system support and strengthening might help identify key collaborators early, which has been recommended as good practice in MDR-TB program development and implementation [55].
